# Complete Photooxidation of Formaldehyde to CO_2_ via Ni-Dual-Atom Decorated Crystalline Triazine Frameworks: A DFT Study

**DOI:** 10.3390/toxics12040242

**Published:** 2024-03-26

**Authors:** Zhao Lu, Zhongliao Wang

**Affiliations:** 1 HKUST Shenzhen-Hong Kong Collaborative Innovation Research Institute, Shenzhen 515100, China; zhaolu@ust.hk; 2 Research and Development Center, Shenzhen Foundation Engineering Co., Ltd., Shenzhen 515100, China; 3 Anhui Province Industrial Generic Technology Research Center for Alumics Materials, Anhui Province Key Laboratory of Pollutant Sensitive Materials and Environmental Remediation, School of Physics and Electronic Information, Huaibei Normal University, Huaibei 235000, China

**Keywords:** CTFs, dual atoms, formaldehyde oxidation, DFT calculation, structural design

## Abstract

Formaldehyde (CH_2_O) emerges as a significant air pollutant, necessitating effective strategies for its oxidation to mitigate adverse impacts on human health and the environment. Among various technologies, the photooxidation of CH_2_O stands out owing to its affordability, safety, and effectiveness. Nitrogen-rich crystalline triazine-based organic frameworks (CTFs) exhibit considerable potential in this domain. Nevertheless, the weak and unstable CH_2_O adsorption hinders the overall oxidation efficiency of CTF. To address this limitation, we incorporate single and dual Ni atoms into nitrogen-rich CTFs by density functional theory (DFT) calculations, resulting in CTF-Ni and CTF-2Ni. This strategic modification significantly enhances the adsorption capability of CH_2_O. Notably, this synergy between Ni dual atoms activates CH_2_O by strong chemical adsorption, thereby reducing the energy barrier of CH_2_O oxidation and achieving the complete oxidation of CH_2_O to CO_2_. Moreover, the introduction of dual-atom Ni over CTF ameliorates visible and near-infrared light absorption and facilitates photoexcited charge transfer and separation. Finally, the underlying mechanisms of complete CH_2_O oxidation over CTF-2Ni are proposed. This work offers novel insights into the rational design of photocatalysts for CH_2_O oxidation through the integration of Ni dual atoms into CTFs.

## 1. Introduction

Indoor formaldehyde (CH_2_O) pollution, primarily resulting from construction and renovation materials, poses significant threats to human health [1,2,3]. Consequently, employing green and eco-friendly methods for the adsorption, transformation, or degradation of CH_2_O is crucial for attaining indoor air quality standards and protecting human health [4,5,6]. Among various CH_2_O remediation technologies, the photocatalytic oxidation of CH_2_O, with its green, economical, and efficient characteristics, emerges as a promising means to mitigate the hazards of CH_2_O [7,8]. Numerous inorganic semiconductors, such as TiO_2_ [9,10,11,12], BiVO_4_ [13], g-C_3_N_4_ [14], ZnO [15], single-atom catalysts [16], and metal–organic frameworks (MOFs) [17,18], have been applied in the research on CH_2_O photooxidation. Despite these materials displaying certain levels of CH_2_O degradation performance, the efficient degradation of CH_2_O over inorganic semiconductors is hindered by the severe recombination of photogenerated charges [19,20,21,22]. Additionally, their complex structures make the elucidation of mechanisms behind CH_2_O adsorption and oxidation challenging [23,24].

Covalent organic frameworks (COFs) with well-defined units, high surface areas, and abundant porous structure, have garnered extensive attention for enhancing CH_2_O oxidation performance and elucidating oxidation mechanisms [25,26,27]. The composition of COFs primarily consists of light non-metallic elements, wherein the atoms are interconnected through covalent bonds to ensure the coordination saturation of these atoms [28]. While the high surface area of COFs facilitates enhancing the adsorption capacity for CH_2_O, the interaction between CH_2_O molecules and COFs is predominantly physical adsorption, which is driven mainly by weak van der Waals forces [29,30,31,32]. This leads to difficulties in the activation and transformation of adsorbed CH_2_O molecules with adsorption easily reaching saturation. Under certain conditions, such as high temperature and pressure, these physically adsorbed CH_2_O molecules may be released, leading to secondary pollution. Common modification methods for COFs, such as heterojunction composite and ligand adjustment, improve their oxidation capabilities to a certain extent [32,33,34,35]. However, these schemes primarily enhance the light absorption and charge separation without fundamentally addressing the adsorption mode of CH_2_O molecules to form stable chemical adsorption, thus failing to achieve the deep activation of CH_2_O molecules and reduce the energy barrier for their oxidative decomposition [36].

Considering the abundant d-orbital electrons in transition metals, which facilitate bonding with gas molecules in comparison to pristine organic COFs, they are more prone to forming chemical adsorption with CH_2_O molecules [37,38]. Therefore, incorporating transition metals into COFs to enhance the chemical adsorption of CH_2_O molecules represents a viable strategy. The exceptional catalytic performance of COFs can be achieved by loading single atoms, which maximizes the preservation of their high surface area, porous structure, and atomic utilization efficiency of transition metals [39]. Although single-atom loading can improve adsorption capacity and molecule activation, the catalytic transformation of small gas molecules by single atoms is generally incomplete with activity still in need of enhancement [40]. Thus, embedding dual-atom catalysts in COFs presents a substantial opportunity to surpass the constraints of single-atom catalysts. However, the precise design and successful construction of dual-atom catalyst loading sites on COFs remain significant experimental challenges with few successes [16]. Thus, the strategic design of dual-atom photocatalysts becomes imperative to direct experimental synthesis and unveil the underlying mechanisms of CH_2_O photooxidation.

In this study, nickel was introduced in both single and dual atoms into a covalent triazine framework (CTF) enriched with pyridinic nitrogen, resulting in the CTF-Ni and CTF-2Ni variants, and their structural, optical, and catalytic characteristics were investigated theoretically. The impact of nickel integration on CTF’s structure was initially explored through X-Ray Diffraction (XRD) and Fourier Transform Infrared (FT-IR) spectroscopy. The influence of nickel on the optical characteristics was then investigated using Ultraviolet-Visible Diffuse Reflectance Spectroscopy (UV-Vis DRS) and X-ray Absorption Spectroscopy (XAS). The effect of nickel on the internal electric field was examined via dipole moment analysis, and its impacts on the conduction band, valence band, and frontier orbitals were assessed using the band structure, density of states (DOS), and HOMO–LUMO analyses. Charge transfer and separation were analyzed through the work function and charge density difference. The rate-determining step (RDS) and energy barriers in the CH_2_O oxidation process were evaluated by changes in the free energy of adsorbed intermediates. This research aims to serve as a benchmark for the future development of dual-atom photocatalysts in the photooxidation of CH_2_O.

## 2. Computational Details

Density functional theory (DFT) simulations were executed using the Vienna ab initio Simulation Package (VASP), utilizing the projector augmented-wave (PAW) method. The exchange-correlation functional was approached through the generalized gradient approximation (GGA) as proposed by Perdew, Burke, and Ernzerhof (PBE). For surface calculations, the Brillouin zone sampling was conducted with a mesh of 2 × 2 × 1 K-points. The energy cutoff was established at 500 eV. Structural optimizations were pursued until the convergence criteria for energy and force were achieved at less than 1 × 10^−5^ eV and 0.02 eV/Å, respectively. To prevent interactions among periodic surface models, a vacuum spacing of 15 Å was implemented. The consideration of van der Waals (vdW) interactions was enhanced by incorporating the DFT-D3 method with zero damping, as developed by Grimme. The computation of Gibbs free energy, ΔG, was formulated as the sum of the electronic energy change (ΔE), zero-point energy change (ΔE_ZPE_), and the entropy change (ΔS) times temperature (T), explicitly ΔG = ΔE + ΔE_ZPE_ − TΔS. The calculation of zero-point energies for both isolated and adsorbed intermediates was based on frequency analysis. Vibrational frequencies and entropies for gas-phase molecules were conducted by the VASPKIT code [41]. Spectral analyses, including vibration and excited state spectra, were conducted with the CP2K-2023.2 software package, employing the PBE functional for system description. The electronic structure was assessed through unrestricted Kohn–Sham DFT within the Gaussian and plane waves (GPW) approach, using Goedecker–Teter–Hutter (GTH) pseudopotentials and the triple-zeta valence with a two polarization functions (TZV2P-MOLOPT-GTH) basis set for all elements. A plane-wave cutoff of 400 Ry was applied, and geometries were optimized employing the Broyden–Fletcher–Goldfarb–Shanno (BFGS) algorithm. The self-consistent field (SCF) method’s density matrix convergence was set to 1 × 10^−5^ Hartree with the force convergence criterion established at 4.5 × 10^−4^ Bohr per Hartree. The excited states and their corresponding spectra were conducted employing the Multiwfn suite [42].

## 3. Results and Discussion

**Figure 1:**
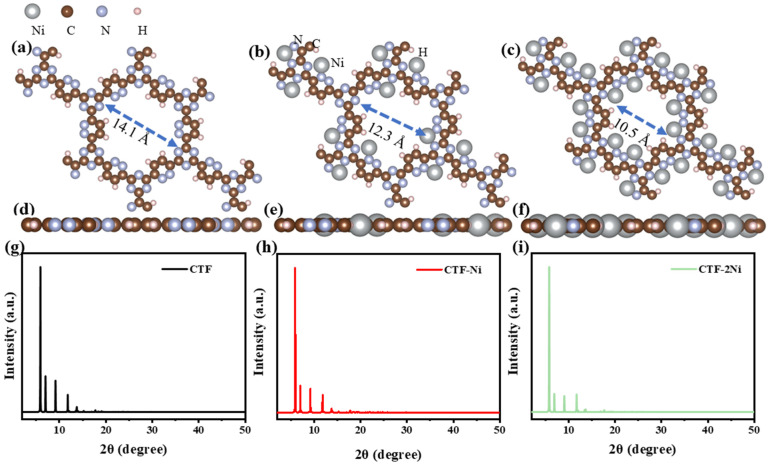
Top view of crystal structure of (**a**) CTF, (**b**) CTF-Ni and (**c**) CTF-2Ni. Side view of crystal structure of (**d**) CTF, (**e**) CTF-Ni and (**f**) CTF-2Ni. Simulated XRD pattern of (**g**) CTF, (**h**) CTF-Ni and (**i**) CTF-2Ni.

The distinct structure of CTF reveals the alternating arrangement and connection of triazine ring (TR) and pyridazine (Figure 1a). For a comprehensive investigation into the efficacy and specific mechanisms of the photocatalytic CH_2_O oxidation by single and dual atoms, we constructed CTF-Ni with Ni single atoms (31.1 wt% Ni) and CTF-2Ni with Ni dual atoms (47.4 wt% Ni). The Ni single atom is loaded onto an N atom of TR and an N atom of the connected pyridazine, forming simultaneous bonds with both N atoms (Figure 1b). In contrast, the other Ni in the Ni dual atoms only bonds with another N atom within the same pyridazine and the N phase on another TR connected to pyridazine (Figure 1c). To elucidate the specific mechanism of single- or dual-atom photocatalytic oxidation of CH_2_O, a single-layer structure was employed for simulation calculations of the three aforementioned materials (Figure 1d–f). The structural diagrams highlight that loading Ni atoms onto the CTF induces a reduction in the pore size. The pore diameter of the pure CTF is 14.1 Å, whereas after loading Ni single atoms (CTF-Ni), the pore diameter decreases to 12.3 Å. With the continued loading of Ni atoms to form CTF-2Ni, the pore diameter further decreases to 10.5 Å, marking a 25% reduction compared to the pure CTF. The XRD simulation calculations reveal that the primary peak of CTFs were observed within the 10° range. Specifically, CTF exhibits three robust peaks at 5.90, 7.00, and 9.15°, respectively. It is worth noting that these three strong peaks of CTF-Ni containing Ni single atoms are also located at 5.90, 7.00 and 9.15°, reflecting the pattern of pure CTF. Similarly, the three prominent peaks of CTF-2Ni align entirely with those of CTF. The XRD results indicate that the incorporation of Ni atoms does not alter the lattice parameters of CTFs, affirming the retention of a highly ordered structure. The simulated structures demonstrate complete uniformity in lattice parameters with a = b = 14.57 Å and c = 15.00 Å for all three types.

In the following analysis, the bond lengths in three CTF materials are investigated to assess how single or dual atoms affect the chemical bonds within the CTF (Table 1). According to previous research findings, the C–N bond length within the isolated TR group is 1.327 Å [25]. However, within the TR group embedded in CTF, the C2–N1 bond length expands to 1.347 Å. This expansion signifies that the TR group is influenced by pyridazine, specifically revealing a repulsive interaction between the N atoms in pyridazine and those within the TR group. This leads to an extension of the C2–N1 bond length within the TR group in CTF. Subsequently, with the loading of Ni single atoms onto CTF, the bond lengths of N1–C2 and C3–N4 experience an increase. This is attributed to the formation of covalent bonds between Ni and N, driven by the overlapping of electron clouds, inducing atomic shifts. Additionally, the larger size of Ni atoms and the generally lower zero-point vibration energy associated with heavier atoms contribute to their reduced susceptibility to vibration during covalent bond formation. Lighter atoms, being more prone to thermal vibrations, may undergo greater deflection during bond formation. Consequently, the N atom shifts toward the Ni atom, resulting in increased bond lengths for N1–C2 and C3–N4. However, the bond length of N4–N5 decreases when a single Ni atom is loaded, which shows that the N atom bonded to Ni on pyridazine moves toward another N on pyridazine under the influence of Ni. However, when Ni atoms continued to be added to CTF-Ni to form a Ni diatomic structure, it was found that the bond lengths of N1–Ni6 and N4–Ni6 both decreased. This shows that the dual-atoms configuration of Ni is more stable than a single Ni atom. Similarly, the N4–N5 bond length in CTF-2Ni only increases slightly, indicating that the addition of a single Ni atom has made the N4–N5 bond of pyridazine in a stable state. This means that dual atoms do not significantly affect the bond lengths within the CTF. In summary, the introduction of Ni atoms will affect the bond length inside CTF to a certain extent, thereby significantly changing the light absorption of CTF and affecting the photooxidation ability of CTF for CH_2_O pollution.

**Figure 2:**
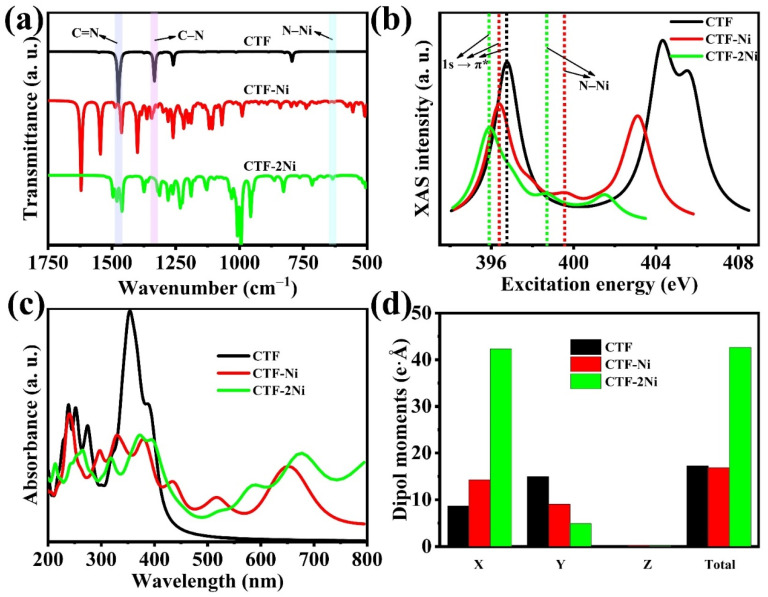
(**a**) Simulated IR spectra of CTF, CTF-Ni and CTF-2Ni. (**b**) Simulated synchrotron X-ray absorption spectra of the N K edge of CTF, CTF-Ni and CTF-2Ni. (**c**) Simulated UV-Vis spectra of CTF, CTF-Ni and CTF-2Ni. (**d**) Dipole moments on different components of CTF, CTF-Ni and CTF-2Ni.

The infrared spectroscopy relies on the vibrational motion of atoms within molecules, encompassing modes like stretching, bending, and twisting. Atoms within a molecule engage in relative vibrational motions, and these vibrations closely correlate with the molecule structure and bond characteristics. These principles allow for using DFT simulations to describe electronic structural and molecular vibrations within a quantum mechanical framework. In the simulated infrared spectrum of CTF, characteristic absorption peaks of TR are observed at wave numbers 1331 and 1476 cm^−1^ (Figure 2a). Specifically, the 1331 cm^−1^ peak corresponds to the stretching vibration mode of C–N in TR, while the 1476 cm^−1^ peak corresponds to the stretching vibration mode of C=N in TR. For CTF-Ni with the incorporation of a single Ni atom, characteristic absorption peaks belonging to TR are retained. The 1345 cm^−1^ peak corresponds to the stretching vibration mode of C–N in TR, while the 1487 cm^−1^ peak corresponds to the stretching vibration mode of C=N in TR. However, in comparison to CTF, the peak intensity of the TR-associated absorption peak in CTF-Ni is significantly weakened, indicating that the presence of Ni single atoms strongly influences the TR vibration in CTF. Similarly, in CTF-2Ni, the characteristic absorption peak associated with TR is notably weakened. However, with the introduction of single Ni atoms, a new peak at 629 cm^−1^ emerges in CTF-Ni, representing the bending vibration peak of Ni–N. Notably, as the Ni loading increases, the bending vibration peak at 632 cm^−1^ belonging to Ni–N in CTF-2Ni becomes much stronger than that in CTF-Ni.

XAS revolves around the absorption of inner shell electrons in materials when the energy of incident X-rays aligns with the energy level of specific element inner-shell electrons. DFT provides a precise description of these core electrons, rendering it a potent tool for simulating XAS. Given the lone pair of electrons on N, facilitating its effective combination with metal Ni to form a Ni single/dual-atom structure, XAS simulation calculations are executed for the N sites in CTFs materials (Figure 2b). This aims to elucidate the chemical environment surrounding the N sites in different CTFs. In CTF, a substantial absorption peak at 396.76 eV corresponds to 1s → π* of N. Specifically, the 1s orbital of N serves as the inner orbital, while the π orbital represents the antibonding orbital linked to the π bond. The XAS absorption edge manifests when the incident X-ray energy aligns with the electronic transition from the 1s orbital to the π orbital of the N atom. Upon the introduction of Ni atoms, the 1s → π* absorption peak of N in CTFs starts shifting toward lower energy. This shift signifies a reduction in the oxidation state of the N atom, indicating the acquisition of electrons by the N atom when bonding with Ni. The appearance of a peak at 399.56 eV in CTF-Ni, absent in CTF, is attributed to a newly formed electronic excited state resulting from electron transfer from N to Ni during their bonding. Notably, the peak in CTF-2Ni, positioned at 398.57 eV, is shifted toward lower energy compared to CTF-Ni. This shift indicates a lower oxidation state of N on the N–Ni bond in CTF-2Ni, promoting the stability of the N–Ni bond and enabling Ni to consistently participate in catalytic reactions.

Utilizing DFT for electron transition calculations and employing the Kubo–Greenwood formula to compute the UV-Vis absorption spectrum, we investigated the impact of Ni atom loading on the light absorption properties of CTF (Figure 2c). Initially, CTF exhibits two prominent absorption peaks at 210–299 nm and 308–450 nm, respectively. The peaks at 210–299 nm may be associated with intramolecular electron transfer, while those in the 308–450 nm range, within the blue light region, may be linked to diverse electronic excitation processes. Notably, the absorption edge of CTF is around 450 nm, indicating an insufficient absorption of visible light. Upon loading Ni atoms onto CTF to form CTF-Ni, there is a notable extension of the absorption range to 450–750 nm. The absorption edge of CTF-Ni reaches 750 nm. This extension may be attributed to the interaction between the 3d orbital of the Ni atom and the orbital of the N atom, causing an adjustment in the energy level structure of the entire system. Simultaneously, the formation of new chemical bonds, such as Ni-N bonds, leads to charge transfer processes, which are observable in the UV-Vis spectrum. Continuing to load Ni atoms into CTF-Ni to create CTF-2Ni with Ni dual atoms, it is observed that the absorption edge of CTF-2Ni extends to 1000 nm, nearly reaching the infrared region. Consistently loading Ni atoms onto CTF to form Ni single/dual atoms enhances the light absorption characteristics of CTF.

The dipole moment magnitudes of the three single-layer CTFs materials were computed using DFT to illustrate the asymmetry of the positive and negative charge distribution within the CTFs materials (Figure 2d). Firstly, the dipole moment has an impact on the absorption spectrum of electromagnetic radiation. Molecules with larger dipole moments are generally more prone to absorbing light in the visible or near-infrared spectral range, contributing to enhanced photocatalytic activity. Notably, CTF-2Ni exhibits the largest dipole moment, suggesting that it could possess the broadest absorption edge. This observation aligns with the findings from the previously calculated UV-Vis spectrum. Secondly, a larger dipole moment is typically associated with an uneven charge distribution within CTFs. CTF-2Ni with the largest dipole moment possesses the strongest built-in electric field, thus facilitating the transfer of photoexcited charge. This implies that during the photocatalytic reaction process, CTF-2Ni may possess the lowest recombination efficiency of photogenerated carriers.

**Figure 3:**
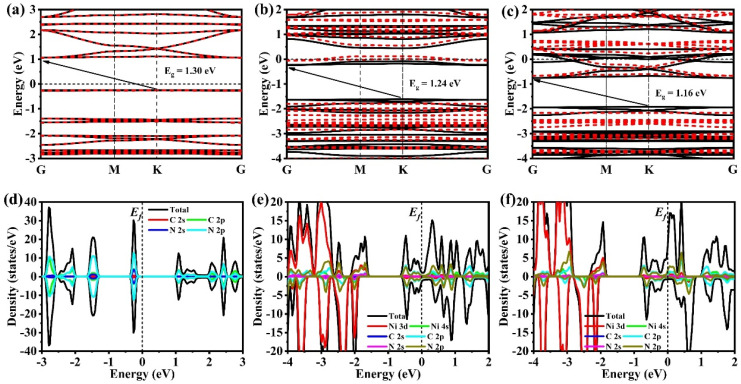
Band structure of (**a**) CTF, (**b**) CTF-Ni and (**c**) CTF-2Ni. Density of states (DOS) of (**d**) CTF, (**e**) CTF-Ni and (**f**) CTF-2Ni.

To achieve a more profound understanding of the influence of loaded Ni single/dual atoms on the electronic structure of CTFs, the band structure and state density of the three simulated CTF materials were calculated. After a detailed analysis of the energy band structure diagrams for the three CTF materials, it was found that all of them have indirect band gaps. Notably, CTF has the widest band gap of 1.30 eV (Figure 3a–c). It is clear that as the concentration of Ni atoms in CTF increases, the band gap decreases. Specifically, CTF-2Ni has the smallest band gap at 1.16 eV. This result indicates that low-energy light is sufficient to excite electrons in CTF-2Ni from the HOMO to the LUMO, facilitating their participation in the oxidation of CH_2_O. Simultaneously, the reduced band gap in CTF-2Ni corresponds to an expanded light absorption edge, indicating that CTF-2Ni can capture sunlight more efficiently. Incorporating Ni single atoms into CTF shifts the Fermi level (E_f_) from the valence band to the conduction band, resulting in the formation of n-type degenerate semiconductors (Figure 3d,e). This observation indicates that by incorporating Ni single atoms, CTF transitions toward a metal-like semiconductor, thereby improving its light absorption efficiency. Notably, the Fermi level of CTF-2Ni extends further into the conduction band compared to that of CTF-Ni, indicating that CTF-2Ni possesses a higher charge transfer efficiency. Furthermore, the specific effects of Ni loading on the density of states of CTFs are examined (Figure 3d–f). The valence band of CTF is primarily contributed by the 2s and 2p orbitals of N, whereas the conduction band of CTF is contributed by the 2p orbitals of C and N. This phenomenon arises from the distinct electronic structures of C and N. It is noteworthy that in CTF-Ni, the 3d orbital of Ni significantly contributes to the composition of the valence band owing to the coordinate covalent bond between N 2p and Ni 3d orbitals. With the further formation of the diatomic CTF-2Ni, the contribution of Ni 3d orbitals to the valence band becomes more obvious.

**Figure 4:**
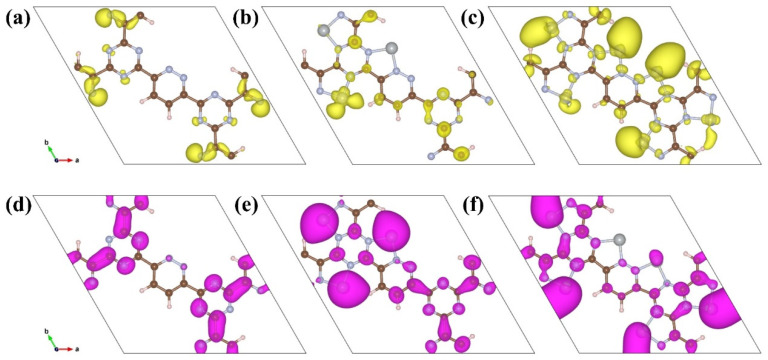
Top view of the HOMO of (**a**) CTF, (**b**) CTF-Ni, (**c**) CTF-2Ni and the LUMO of (**d**) CTF, (**e**) CTF-Ni, (**f**) CTF-2Ni.

To demonstrate the effect of loading Ni single or dual atoms on the electronic structure of CTFs, 2D top views of the HOMO and LUMO bands for three CTF materials are displayed. For pristine CTF, the HOMO energy mainly locates on the nitrogen atoms in pyridazine and adjacent carbon atoms (Figure 4a). Nitrogen on triazine rings also slightly contributes to the HOMO bands, aligning with the density of states of CTF. The LUMO band distribution covers carbon and nitrogen in both triazine and pyridazine (Figure 4d). Introducing Ni single atoms into CTF modifies the HOMO and LUMO bands significantly, reflecting the strong interaction of the metallic Ni with CTF (Figure 4b,e). Part of the HOMO bands localizes on single Ni atoms, and the rest is on triazine. Notably, a large part of the LUMO bands centers on Ni atoms. CTF-2Ni presents notable changes in its HOMO and LUMO bands relative to CTF-Ni, further underscoring that Ni can effectively tune frontier orbitals and active sites (Figure 4c,f). In CTF-2Ni, Ni atoms are the main contributors to both HOMO and LUMO bands. This highlights the critical role of dual-atom Ni in CTF-2Ni for the modulation of electrical structure and active sites for CH_2_O oxidation.

**Figure 5:**
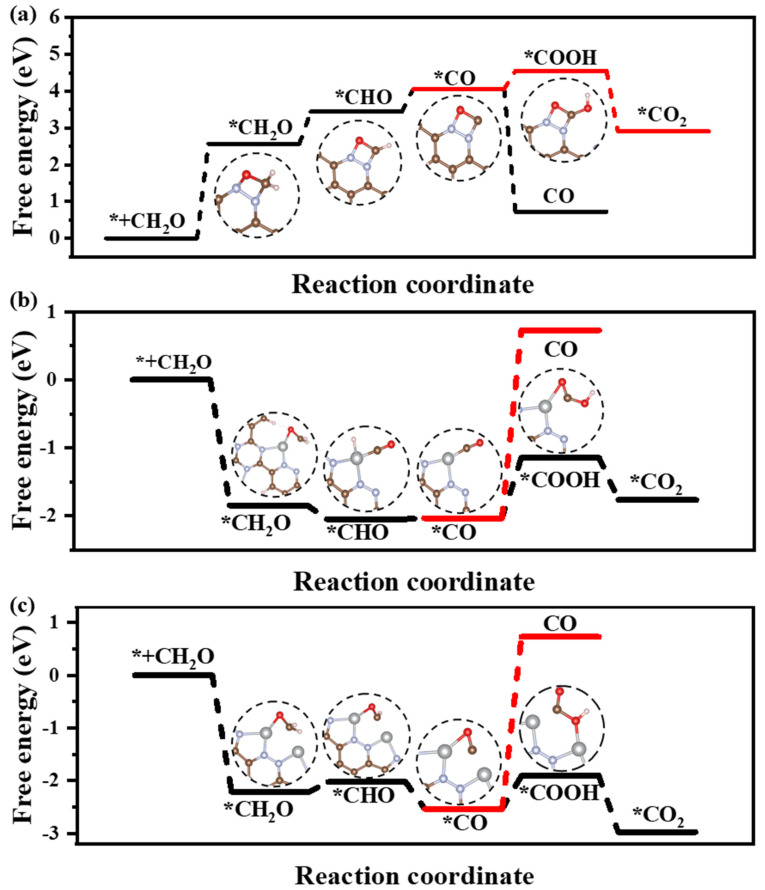
Free-energy diagrams and key intermediates during the CH_2_O oxidation for the CH_2_O oxidation to CO or CO_2_ on the (**a**) CTF, (**b**) CTF-Ni and (**c**) CTF-2Ni.

Catalytic formaldehyde oxidation (CFO) calculation results reveal that the rate-determining step (RDS) for CFO on CTF is the adsorption of CHO on CTF (* + CH_2_O = *CH_2_O) with an energy barrier of 2.57 eV (Figure 5a). The incorporation of Ni atoms significantly enhances the adsorption energy of CTF for CH_2_O molecules. The negative adsorption free energy of CH_2_O on CTF-Ni and CTF-2Ni suggests that the introduction of Ni enables CTF to spontaneously adsorb CH_2_O from the indoor or external environment (Figure 5b,c). The adsorption free energy of CTF-2Ni shows a stronger negativity, indicating an improved ability to adsorb CH_2_O. Furthermore, the intermediates during the CH_2_O oxidation on CTF, CTF-Ni and CTF-2Ni are displayed to illustrate the significance of Ni for the oxidation process of CH_2_O and decreased energy barrier (Table 2). Initially, CH_2_O is adsorbed on CTF through O4–N5 and C3–N6 bonds with the two N atoms on the pyridazine, respectively. However, the C3–O4 bond in CH_2_O is stretched to 1.451 Å, which is not conducive to the catalytic oxidation of CH_2_O to CO_2_ in CTF. Furthermore, the low adsorption capacity of CTF causes the adsorbed CO to desorb easily, resulting in incomplete CH_2_O oxidation (Figure 5a). However, incomplete oxidation of CH_2_O molecules into CO remains detrimental to human health. The integration of Ni modifies the rate-determining step in the CFO process by chemically linking the oxygen in CH_2_O to the Ni atom, enabling Ni-enriched CTF to adsorb CH_2_O molecules spontaneously (Figure 5b,c). Moreover, with CTF-2Ni having twice the Ni content of CTF-Ni, CTF-2Ni can provide more active sites and strong adsorption capability. In addition, with the introduction of Ni, the RDS of CFO changes into the conversion of *CO to *COOH (*CO + H_2_O + h^+^ = *COOH + H^+^). Finally, *COOH is completely oxidized to CO_2_ under the influence of photogenerated holes. Furthermore, the reaction energy barriers corresponding to the introduction of Ni single atoms and double atoms on CTF are 0.89 and 0.63 eV, respectively. This result indicates that diatomic Ni is thermodynamically more favorable for oxidizing CH_2_O molecules to CO_2_ than a Ni single atom. In summary, Ni diatoms not only facilitate the adsorption and complete oxidation of formaldehyde molecules but also significantly reduce the reaction energy barrier for CH_2_O oxidation.

* + CH_2_O = *CH_2_O (1)

*CH_2_O + h^+^ = *CHO + H^+^ (2)

*CHO + h^+^ = *CO + H^+^ (3)

*CO + H_2_O + h^+^ = *COOH + H^+^ (4)

*COOH + h^+^ = *CO_2_ + H^+^ (5)

*CO = * + CO(6)

**Figure 6:**
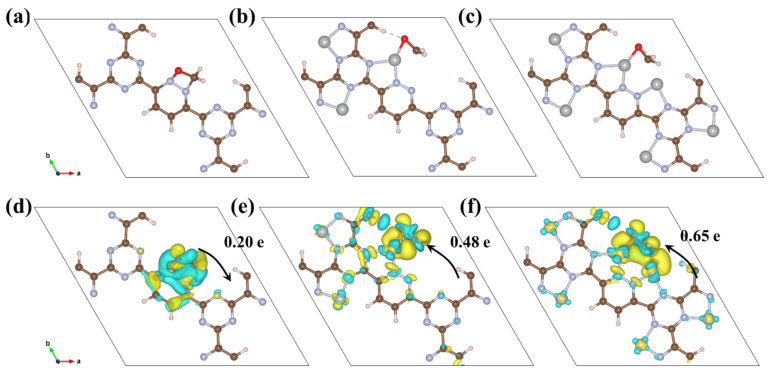
Top view of the optimized (**a**) CTF, (**b**) CTF-Ni, (**c**) CTF-2Ni. Top view of the charge density difference of (**d**) CTF, (**e**) CTF-Ni, and (**f**) CTF-2Ni adsorbed with CH_2_O with an isosurface of 2 × 10^–3^ e/Å^3^ (charge accumulation is marked in yellow, while reduction is in cyan).

To further analyze the direction and quantity of electron transfer in the three CTFs after adsorbing CH_2_O, Bader charge analysis was conducted. After CH_2_O adsorption on CTF, 0.2 electrons transfers from CH_2_O to CTF (Figure 6a,d). Upon loading Ni onto CTF, CH_2_O chemically bonds with Ni on CTF-Ni. However, the electron transfer direction is altered, and 0.48 electrons transfers from CTF-Ni to CH_2_O (Figure 6b,e). CTF-2Ni transfers the most electrons to CH_2_O with 0.65 electrons transfers from CTF-2Ni to CH_2_O (Figure 6c,f). These results demonstrate that the introduction of Ni atoms not only changes the direction of charge transfer between CTF and CH_2_O but also alters the quantity of transferred electrons. During adsorption, the more electrons that transfer between the substrate and the adsorbed substance, the more favorable the adsorption. The Bader charge analysis shows that CH_2_O is most readily adsorbed on CTF-2Ni, whereas CTF offers the least favorable conditions for CH_2_O adsorption. The values of the d band centers of CTF-Ni and CTF-2Ni also give the reason why CTF-2Ni has strong adsorption of CH_2_O. The d band center of diatomic Ni moves downward compared to single-atom Ni, which results in fewer electrons filling the antibonding orbital of Ni 3d (Figure 3e,f). This is a critical factor regarding why diatomic Ni has a stronger ability to adsorb CH_2_O than single-atom Ni. Interestingly, the loading of Ni atoms alters the direction of electron transfer, which is key for the adsorption and activation of CH_2_O. In CTF, electron transfer from CH_2_O to CTF creates an inherent electric field (IEF) oriented from CH_2_O toward CTF. This IEF, however, drives photoexcited holes from CH_2_O to CTF, hindering CH_2_O oxidation. The introduction of Ni reverses the direction of IEF, thus facilitating more photoexcited holes to engage in the oxidation processes of CH_2_O.

**Figure 7:**
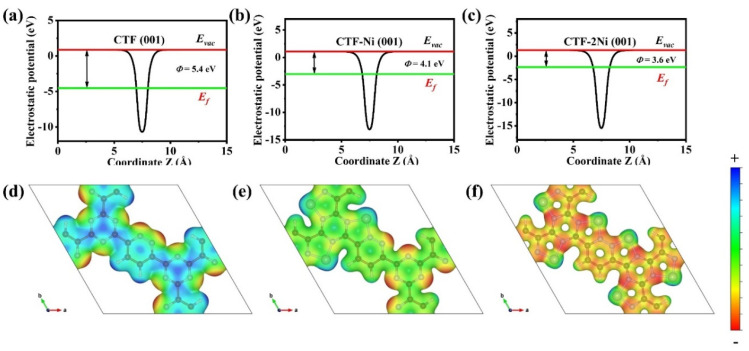
Electrostatic potentials of (**a**) CTF (001) surface, (**b**) CTF-Ni (001) surface and (**c**) CTF-2Ni (001) surface. Electrostatic potential diagram of (**d**) CTF, (**e**) CTF-Ni and (**f**) CTF-2Ni.

The work functions of the CTF (001), CTF-Ni (001), and CTF-2Ni (001) surfaces are calculated at 5.4, 4.1, and 3.6 eV, respectively, demonstrating that the incorporation of Ni leads to a reduction in work function values (Figure 7a–c). Moreover, CTF-2Ni with the smallest work function may donate more electrons to adsorbed CH_2_O and elevate CH_2_O oxidation efficiency. This effect primarily arises from Ni acting as an electron donor to CTF, resulting in an upward shift of the Fermi level. The surface electrostatic potentials of the three CTFs offer further insights into the mechanism behind the electron reversal phenomenon triggered by the addition of Ni atoms (Figure 7d–f). The potential at the N-N bond position of CTF is low (Figure 7d). Introducing Ni leads to electron transfer from Ni to the N-N bridge, elevating the potential at the Ni site (Figure 7e,f). However, the electrostatic potential at the N–N bridge and Ni site are opposite, leading to a reversed electron transfer after CH_2_O is adsorbed. As the number of Ni atoms increases, the overall potential of the CTF decreases further. The electrostatic potential of the N-N bridge in pyridazine is notably lower in CTF-2Ni compared to CTF-Ni, accounting for increased electron transfer from CTF-2Ni to adsorbed CH_2_O.

## 4. Conclusions

In summary, this work explores the photooxidation of CH_2_O over pristine CTF and CTF embedded with single and dual Ni configurations. First, incorporating Ni into CTF slightly reduces the band gap and induces a modest redshift of absorption edge, thereby facilitating visible and near-infrared light absorption. In addition, the introduction of Ni lowers the work function and elevates the Fermi level, consequently reversing photoexcited charge transfer compared with pristine CTF and driving the hole from the CTF-2Ni to CH_2_O to be involved in photooxidation processes. Moreover, the introduction of Ni intensifies the dipole moment of CTF-2Ni and induces a strong IEF, boosting photoexcited charge separation. More importantly, the strong chemical adsorption and increased charge transfer of CH_2_O over CTF-2Ni endow CH_2_O with sufficient activation, thus decreasing the energy barrier of CH_2_O photooxidation and completely converting CH_2_O to CO_2_. This work provides a reference for designing dual-atom covalent organic frameworks for the complete photooxidation of CH_2_O.

## Figures and Tables

**Table 1:** Bond length of CTF, CTF-Ni and CTF-2Ni.

Bond Length (Å)	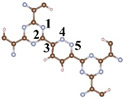	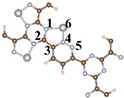	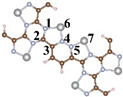
N1–C2	1.347	1.378	1.390
C2–C3	1.524	1.465	1.453
C3–N4	1.357	1.399	1.398
N4–N5	1.335	1.324	1.326
N1–Ni6	-	2.124	1.946
N4–Ni6	-	1.813	1.804
Ni6–Ni7	-	-	3.446

**Table 2:** Bond length and angle distribution of CH_2_O of CH_2_O, CTF, CTF-Ni and CTF-2Ni.

Structure	Bond Length (Å)						Bond Angle (°)		
	C3–H1	C3–H2	C3–O4	N5–O4	N6–C3	Ni7–O4	H1–C3–H2	H1–C3–O4	H2–C3–O4
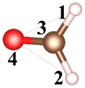	1.118	1.118	1.215	-	-	-	116.15	121.94	121.911.1
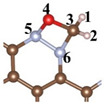	1.103	1.095	1.451	1.573	1.466	-	111.84	111.51	113.59
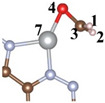	1.105	1.105	1.338	-	-	1.857	115.45	118.59	118.47
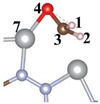	1.11	1.191	1.343	-	-	1.863			

## Data Availability

All the data are available within the manuscript.
